# Ecosystem Processes and Nitrogen Export in Northern U.S. Watersheds

**DOI:** 10.1100/tsw.2001.328

**Published:** 2001-11-16

**Authors:** Robert Stottlemyer

**Affiliations:** U.S. Geological Survey, Ft. Collins, CO 80526, USA

**Keywords:** nitrogen budget, watershed, northern ecosystem, national park, Michigan, Colorado, Alaska

## Abstract

There is much interest in the relationship of atmospheric nitrogen (N) inputs to ecosystem outputs as an indicator of possible “nitrogen saturation” by human activity. Longer-term, ecosystem-level mass balance studies suggest that the relationship is not clear and that other ecosystem processes may dominate variation in N outputs. We have been studying small, forested watershed ecosystems in five northern watersheds for periods up to 35 years. Here I summarize the research on ecosystem processes and the N budget. During the past 2 decades, average wet-precipitation N inputs ranged from about 0.1 to 6 kg N ha year among sites. In general, sites with the lowest N inputs had the highest output-to-input ratios. In the Alaska watersheds, streamwater N output exceeded inputs by 70 to 250%. The ratio of mean monthly headwater nitrate (NO_3_) concentration to precipitation NO_3_ concentration declined with increased precipitation concentration. A series of ecosystem processes have been studied and related to N outputs. The most important appear to be seasonal change in hydrologic flowpath, soil freezing, seasonal forest-floor inorganic N pools resulting from over-winter mineralization beneath the snowpack, spatial variation in watershed forest-floor inorganic N pools, the degree to which snowmelt percolates soils, and gross soil N mineralization rates.

## INTRODUCTION

There is much interest in the relationship of atmospheric nitrogen (N) inputs to ecosystem outputs as an indicator of possible "nitrogen saturation" by human activity[1]. Long-term, ecosystem-level mass balance studies show that the relationship is not clear and that other ecosystem processes may account for the variation in N outputs[2]. In forested and alpine ecosystems, processes such as canopy retention of N, soil microbial immobilization, change in seasonal hydrologic flowpath, and climatic factors such as soil freeze-thaw frequency can be important[3,4]. However, such factors are rarely considered in discussions of ecosystem N budgets.

In 1982, the U.S. National Park Service established a network of watershed ecosystems to undertake long-term study of ecosystem structure and function and their response to atmospheric chemical inputs and other components of global change. Most of the original sites were northern systems. Since then, the network has been expanded by addition of several southern U.S. ecosystems.

Here I summarize published, longer-term study results from five of the northern sites located in Michigan, Colorado, and Alaska (Table 1). Asik, Rock Creek, and Lexen Creek watersheds have both alpine and subalpine ecosystems. The Wallace Lake and Calumet watersheds are at the ecotone between the northern hardwoods and southern boreal forest. The Calumet and Wallace Lake watersheds have moderate atmospheric N inputs and Lexen Creek low N inputs, while Rock Creek and Asik watersheds have some of the lowest atmospheric N inputs in North America. The N deposition gradient across the sites was 50-fold (Table 2). The sites have similar databases that are useful to examine possible processes regulating ecosystem retention and loss of atmospheric N inputs. My objectives here are to (1) quantify relationships between atmospheric N inputs and headwater and subalpine streamwater nitrate ($NO_3^-$) concentrations and flux, and (2) to better define the physical and biogeochemical processes regulating ecosystem N retention and release.

**TABLE 1**
**Site, Latitude and Longitude, Elevation, Size, Temperature, Onsite Precipitation, Soil Taxonomy, Geology, and Vegetation of the NPS–USGS Long-Term Watershed Ecosystem Study Sites[a]**

| Watershed Site Name | Lat. (N) Long. (W) | Elev. (M) | Watershed Size (ha) | Temp. Jan. and July (°C) | Precip. (cm) | Soils | Geology | Vegetation |
|---|---|---|---|---|---|---|---|---|
| Calumet, Upper Peninsula, Michigan | 47° 48′ 88° 15′ | 190–375 | 176 | –9 and 16 | 87 | Alkaline glacial till and old beach deposits | Precambrian Portage Lake volcanics | Sugar maple (80), white birch |
| Wallace Lake, Isle Royale NP, Michigan | 47° 48′ 88° 15′ | 184–425 | 115 | –9 and 16 | 75 | Reworked postglacial beachline and sand deposits | Metamorphosed flood basalts, alkaline till | Aspen, white birch, white spruce (160) |
| Lexen Creek, Fraser Exper. Forest, Colorado | 39° 54′ 105° 53′ | 2980–3515 | 124 | –11 and 13 | 102 | Gravel, sandy loam, alluvium, carbonates | Gneiss, schist sedimentary sandstone | Aspen, willow, lodgepole pine, spruce (300), subalpine fir |
| Rock Creek, Denali NP and Preserve, Alaska | 63° 44′ 149° 00′ | 625–1737 | 770 | –21 and 15 | 41 | Permafrost, young soil from weathered bedrock | Weathered schist and gneiss | Tundra, moss, spruce, birch, *Populus*, willow, sedge, cottongrass |
| Asik, Noatak National Preserve, Alaska | 67° 58′ 162° 15′ | 15–670 | 800 | –22 and 11 | 37 | Alluvial, marine sediments, volcanic ash and loess | Paleozoic sedimentary and metamorphic | Willow, alder, and birch shrub; cottongrass and sedge tussock tundra; white spruce (160) |

[a] Under vegetation, the mean age of the longer-lived canopy species is in parentheses.

## EXPERIMENTAL METHODS/PROCEDURES

The sites considered here were established between 1957 and 1989 (Table 1). Each site consists of a small gauged-watershed, complete year-round meteorological station with lesser-equipped substations, vegetation plots, and lysimeter plots[5,6,7,8]. At most sites, intensive surface-water chemical analyses have been conducted for the last 2 decades. Upstream and downstream surface-water and precipitation chemistry has been monitored year-round or to the extent that access permits. Surface-water pH, alkalinity, and specific conductance, and soil inorganic N extractions were conducted at field laboratories. Surface-water ion chemistry and soil-extract analyses were conducted at our laboratory located at the U.S. Forest Service Rocky Mountain Research Station, Ft. Collins, CO. The level of detection for $Ca^{2+}$ was 0.02 mg $l^{-1}$, $NH_4^+$ 0.01 mg $l^{-1}$, and $NO_3^-$ 0.01 mg $l^{-1}$. Dissolved organic carbon (DOC) and nitrogen (DON) analyses were conducted at Michigan Technological University, Houghton, MI. Isotope analyses (gross N mineralization, immobilization) were conducted at the University of California, Davis. Details of re-

**TABLE 2**
**Precipitation and Streamwater $NH_4^+$-N and $NO_3^-$-N Concentrations (mg N $l^{-1}$), Precipitation Inputs and Streamwater Outputs (kg N $ha^{-1}$ $year^{-1}$), and Input/Output Ratios — NPS–USGS and U.S. Forest Service Fraser Experimental Forest Long-Term Watershed Ecosystem Study Sites**

| | $NH_4^+$ (mg $l^{-1}$) | $NO_3^-$ (mg $l^{-1}$) | Total Inorg. N (kg $ha^{-1}$ $year^{-1}$) | Input/Output Ratio | Reference |
|---|---|---|---|---|---|
| **Inputs** | | | | | |
| Calumet | 0.26 | 0.24 | 5.90 | | 11 |
| Wallace | 0.21 | 0.25 | 3.50 | | 8 |
| Lexen | 0.06 | 0.08 | 2.40 | | 20 |
| Rock Cr. | 0.07 | 0.03 | 0.12 | | 5,21 |
| Asik | 0.05 | 0.04 | 0.30 | | 7 |
| **Outputs** | | | | | |
| Calumet | 0.04 | 0.17 | 0.65 | 9.1 | 11,15 |
| Wallace | 0.04 | 0.08 | 0.80 | 4.4 | 8 |
| Lexen | 0.01 | 0.02 | 0.15 | 10.3 | 6,20 |
| Rock Cr. | 0.04 | 0.28 | 0.31 | 0.4 | 5,21 |
| Asik | 0.03 | 0.21 | 0.50 | 0.6 | 7 |

search procedures and methods used can be found in the literature cited[6,7,8].

## RESULTS AND DISCUSSION

### Climate/Hydrology

Precipitation amounts were highest at the Michigan and Colorado sites and lowest in Alaska (Table 1). There was little seasonal change in precipitation amounts at the Michigan and Colorado sites, but summer precipitation was higher in Alaska (Fig. 1). Snowmelt dominated the annual hydrographs of Calumet, Wallace, and Lexen Creeks, where summer precipitation rarely reaches the stream. The hydrographs for the Alaskan sites were more influenced by late summer and autumn precipitation, late summer thawing of the soil active layer, and the autumn reduction in evapotranspiration. The Asik and Rock Creek watershed hydrographs were "flashy", showing rapid changes in runoff, which is characteristic of watersheds with discontinuous permafrost and extensive seasonally frozen soils. In Alaska, winter freezing of streams makes accurate measurement of discharge, when present, difficult.

All sites were characterized by warming ($p < 0.05$) annual temperatures during the last several decades[9]. The largest increase was in northwestern Alaska (Asik watershed), where mean annual temperatures have increased an average of 0.3°C $year^{-1}$ during the last decade. There has been no trend in annual precipitation amounts for any site.

Soils remained unfrozen beneath the snowpack at Calumet and Wallace, except beneath white spruce (*Picea glauca*) at Wallace, where canopy snowfall interception reduced the insulation capacity of the snowpack. The alpine soils at Lexen and Rock Creek, and subalpine and alpine soils at Asik froze in winter[5,7,8,10]. At Calumet, Wallace, and Lexen the snowpack temperature at 10 cm above the forest floor averaged –3°C, and the snowpack lost a third of its moisture by snowpack peak water equivalent (PWE) through both snowmelt (25%) and sublimation (8%). At Rock Creek and Asik, the snowpack PWE can exceed half the annual precipitation amount.

### Precipitation Ion Input

Volume-weighted precipitation $NH_4^+$ and $NO_3^-$ concentrations varied greater than fivefold among sites (Table 2). Concentrations were greatest at Calumet and Wallace and lowest at Asik and Rock Creek.

Inorganic N inputs were highest (5.9 kg N $ha^{-1}$ $year^{-1}$) at Calumet and lowest (0.12 kg N $ha^{-1}$ $year^{-1}$) at Rock Creek. $NO_3^-$-N was the dominant form of wet-precipitation N input except in Alaska. The small difference in N deposition at the two Alaska sites may not be real since the Asik site has a shorter period of record[7].

At Calumet, up to 50% of annual precipitation inorganic N inputs were temporarily retained in the snowpack[11]. At Wallace the peak snowpack content was about 20%[8] and at Lexen 80% of annual inorganic N inputs[10]. However, by the time of snowpack PWE, all three sites lost half the cumulative winter-precipitation inorganic N inputs. The loss was primarily a function of unfrozen soils, which contribute energy to the base of the snowpack, and of periodic midwinter thaws particularly on more southern aspects.

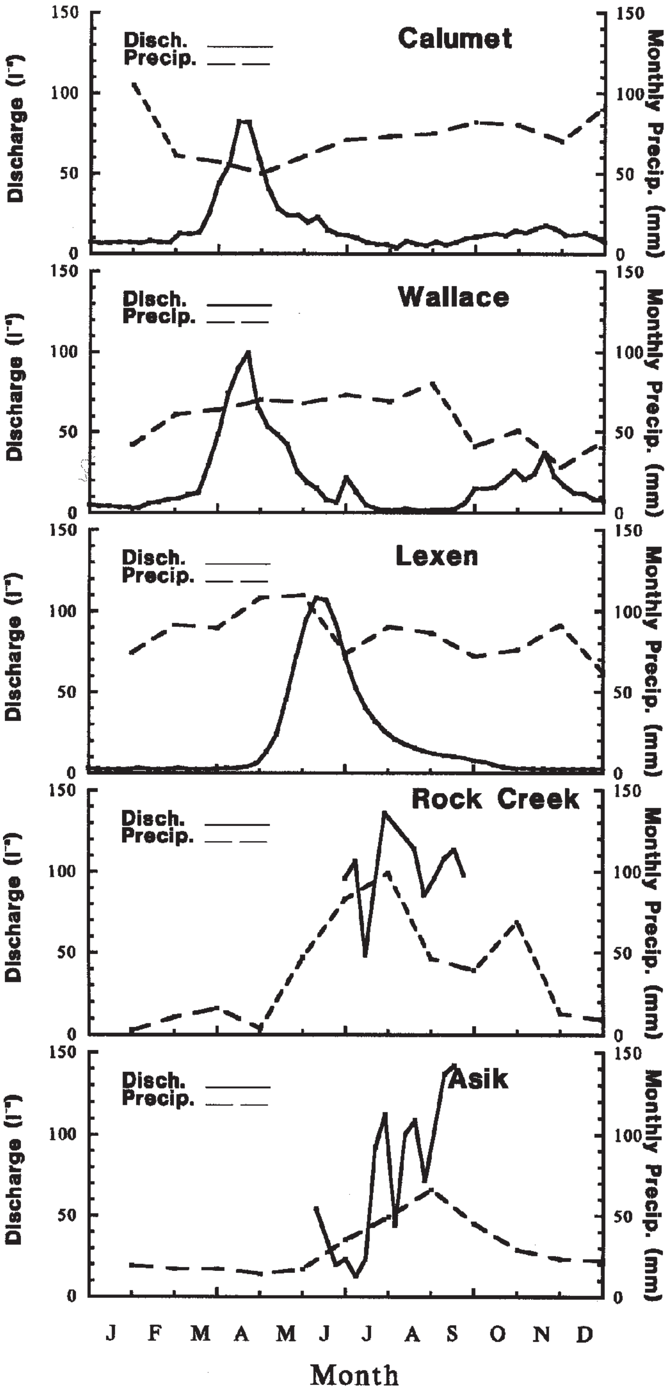

FIGURE 1. Mean seasonal hydrograph and precipitation, Calumet and Wallace Lake, Michigan; Lexen Creek, Colorado; Rock Creek and Asik watersheds, Alaska.

## Streamwater Ion Output

Streamwater $NO_3^-$ and $NH_4^+$ concentrations varied among sites (Table 2) but did not reflect precipitation chemistry. Rock Creek and Asik had the lowest precipitation $NO_3^-$ concentrations but highest streamwater $NO_3^-$ concentrations. In these two watersheds, streamwater $NO_3^-$ concentrations were higher than observed in most North American surface waters, even in regions where atmospheric $NO_3^-$ inputs are elevated. Among all sites, the ratio of mean monthly upstream $NO_3^-$ concentration to precipitation $NO_3^-$ concentration declined ($p < 0.001$, $r^2 = 0.47$) as precipitation $NO_3^-$ concentration increased[6].

Wet-precipitation inorganic N inputs were retained at Calumet, Wallace, and Lexen (Table 2). The strongest retention (>90%) was at Lexen, followed by Calumet. There was no onsite monitoring of dry deposition at any location. Accounting for dry N deposition, as from nitric acid ($HNO_3^-$), ecosystem retention would be still larger. At Asik and Rock Creek, where inputs were <10% of those at the Colorado and Michigan sites, streamwater N output exceeded inputs by 70 to 250% (Table 2), which indicates that they were saturated. Annual streamwater N output at Asik was two thirds or more of the output at Wallace and Calumet, where N inputs were highest.

Except for Calumet, where the forest was dominated by midsuccession hardwoods, all sites were vegetated primarily by old growth forest. Despite the old age of forest vegetation, lack of land-use disturbance, and successional status, atmospheric N inputs were strongly retained except at the Alaska sites. The results from the Alaskan watersheds suggest that there is a minimum, but sizable, inorganic N export from watersheds independent of precipitation inputs[12].

## Factors Regulating Ecosystem N Retention and Release

Calumet had the highest wet-precipitation N inputs but strongly retained inorganic N inputs (Table 2). In a 7-year plot study at Calumet, about 50% of $NO_3^-$-N and 75% of $NH_4^+$-N flux was retained by the canopy and forest floor, and >90% of the atmospheric N inputs were immobilized before reaching the base of the rooting zone[12]. At Wallace and Lexen, atmospheric N inputs were 50 to 60% of the inputs to Calumet. At Wallace, the inorganic N flux in soil water beneath the rooting zone of spruce and birch–aspen was 16% of the atmospheric inputs, a stronger retention than at the watershed level where outputs were 22% of inputs (Table 2). At Lexen Creek, the precipitation amount was greater than at Calumet or Wallace, but atmospheric N inputs were less (Tables 1 and 2). At the watershed level, >90% of atmospheric N inputs were immobilized before reaching the base of the rooting zone. The Lexen subalpine had the strongest N retention of the sites examined here. The Alaskan watersheds, Rock Creek in Denali National Park and Asik in the Noatak National Preserve, had the highest streamwater $NO_3^-$-N concentrations (Figure 3), but atmospheric inorganic N inputs were only 5 to 10% of the inputs at the other sites.

It appears that one factor reducing ecosystem N loss was passage of snowmelt through the forest-floor and surface mineral soils. At Calumet, <10% of snowmelt occurred as overland flow. However, once meltwater entered the forest-floor and surface mineral soil, the snowmelt N content was immobilized. Despite a large snowpack, relatively low forest-floor C:N ratio, and large forest-floor organic N pool, at Calumet it appeared that unfrozen soils permitted strong microbial immobilization of available N throughout the year, as indicated by the low net N mineralization rates (Table 3). At Lexen, snowmelt accounted for >95% of annual stream runoff, but there was little or no overland flow[13]. At Lexen, unfrozen soils and periodic thaws, especially on more southern aspects, resulted in 40 to 45% of the snowpack inorganic N being lost in snowmelt by snowpack PWE[10]. How-

**TABLE 3**
**Forest-Floor (Oe and Oa) Total N Content (kg N ha$^{-1}$), C:N Ratios, and N Mineralization Rates — NPS–USGS and U.S. Forest Service Fraser Experimental Forest Long-Term Watershed Ecosystem Study Sites**

| | N Content (kg N ha$^{-1}$) | C:N | Net N Mineralization (g N ha$^{-1}$ day$^{-1}$) | Gross N Mineralization (g N ha$^{-1}$ day$^{-1}$) | Reference |
|---|---|---|---|---|---|
| Calumet | 1190 | 17 | 3.0 | — | 15 |
| Wallace | 530 | 14 | 38.0 | 882 | 8 |
| Lexen | 730 | 35 | 10.0 | 684 | unpub. data |
| Rock Cr. | 890 | 30 | 0.3 | — | 21 |
| Asik | 1120 | 18 | 30.0 | 4900 | 7 |

ever, almost all inorganic N was immobilized before reaching a 30-cm soil depth.

Canopy retention of atmospheric N inputs, particularly by conifers, was also a factor increasing N retention in these ecosystems. At Wallace, the spruce (*P. glauca*) and birch–aspen (*Betula papyrifera–Populus tremuloides*) canopies, the dominant canopy cover in the watershed, and forest understory retained >50% of wet-precipitation N inputs[8]. At the Fraser Experimental Forest, we have conducted two studies, 10 to 20 years in duration, on the effect of canopy removal on nutrient budgets and loss from the ecosystem[14]. Canopy removal increased the percentage of subsurface flow occurring in shallower soils and disproportionately increased the loss of forest-limiting nutrients as $NO_3^-$ and $K^+$ for more than a decade after treatment. Canopy removal also eliminated winter interception of snowfall. During winter, the subalpine canopy intercepted 0.3 kg N ha$^{-1}$ year$^{-1}$, or about 15% of annual atmospheric N inputs.

In forests where a large percentage of total ecosystem N is in the forest floor and its C:N ratio is less than 20, N mineralization and flux beneath the rooting zone can be large[15,16]. For the sites considered here, most of the ecosystem N was contained within the forest-floor and surface mineral soils[7,8]. The highest percentage of ecosystem N in the forest floor was at Asik (81%) and the lowest at Calumet. There were not sufficient aboveground biomass data for Rock Creek to make a reasonable comparison.

Seasonal change in soil N mineralization rate was also a factor accounting for some differences in ecosystem inorganic N loss. At Calumet, over-winter soil mineralization and other biological processes maintained subsurface ion and DOC reservoirs in shallow soils and the forest floor[17]. Small amounts of snowmelt, which occurred throughout winter, removed the highly mobile $NO_3^-$ from the forest floor, which resulted in elevated winter streamwater concentrations but little flux (Figs. 2 and 3). At Wallace, beneath spruce and birch–aspen the forest-floor net N mineralization rates were relatively high, and gross N mineralization rates were 20 times net rates (Table 3). Both processes suggested large amounts of N cycling in this mature boreal-forested ecosystem. The snowpack PWE inorganic N content averaged 25% of the size of winter inorganic N pools in the forest-floor and surface mineral soil, and forest-floor inorganic N pools were eight times the amount in monthly precipitation N inputs. The relatively small snowpack N content and seasonal precipitation N input compared with the size of forest-floor and shallow-soil inorganic N pools and N mineralization rates, and the strong ecosystem N retention of precipitation N inputs, indicated that little streamwater inorganic N came directly from precipitation. This finding is consistent with results from other small watersheds receiving a gradient of atmospheric N inputs[18]. At Wallace, the peak in streamwater runoff and net forest-floor N mineralization rates occurred in early summer[8]. In the Rock Creek watershed, seasonal forest-floor N mineralization rates and streamwater $NO_3^-$ concentrations peaked at the same time[19]. And at Asik, where streamwater $NO_3^-$ concentrations were high, the net and gross N mineralization rates were some of the highest among sites (Table 3, Fig. 3).

Change in seasonal flowpath of soil water to the stream may be the most significant factor regulating ecosystem N output in these ecosystems. At Calumet, winter and late summer base-flow streamwater $Ca^{2+}$ and other base-cation ($C_B$) and Si concentrations, coupled with shallow-well data, indicated that soil-water levels and flowpaths at base flow were deep (Fig. 2). With snowmelt, soil-water levels and shallow lateral subsurface flow rates quickly rose, diluted $Ca^{2+}$ concentrations, and increased the output of $NO_3^-$ and DOC from the biologically active forest floor. Despite the large snowpack, its $NO_3^-$ content was a minor contribution to spring output[12]. The output of $NO_3^-$ and DOC increased proportionately more than all other ions, especially the $C_B$ and Si, with the gain in stream runoff during snowmelt; and spring output dominated annual inorganic N export. At Wallace, the dominant process regulating streamwater inorganic N export at the watershed level appeared to be hydrologic flowpath, especially during late spring snowmelt[8]. In Lexen Creek, alpine streamwater $Ca^{2+}$ and $NO_3^-$ concentrations changed little during snowmelt, indicating that melt water passed quickly through surface porous soils and was well mixed before entering the stream (Figs. 2 and 3). Conversely, subalpine streamwater $Ca^{2+}$ concentration was diluted >50% during snowmelt, suggesting that much meltwater moved to the stream as shallow subsurface lateral flow. Subalpine streamwater $NO_3^-$ concentrations declined to near detection levels during snowmelt, indicating strong immobilization (Figs. 1 and 3). The experimental studies at the Fraser Experimental Forest on the effect of canopy removal demonstrated that a slight increase in shallow subsurface lateral flow dispropor-

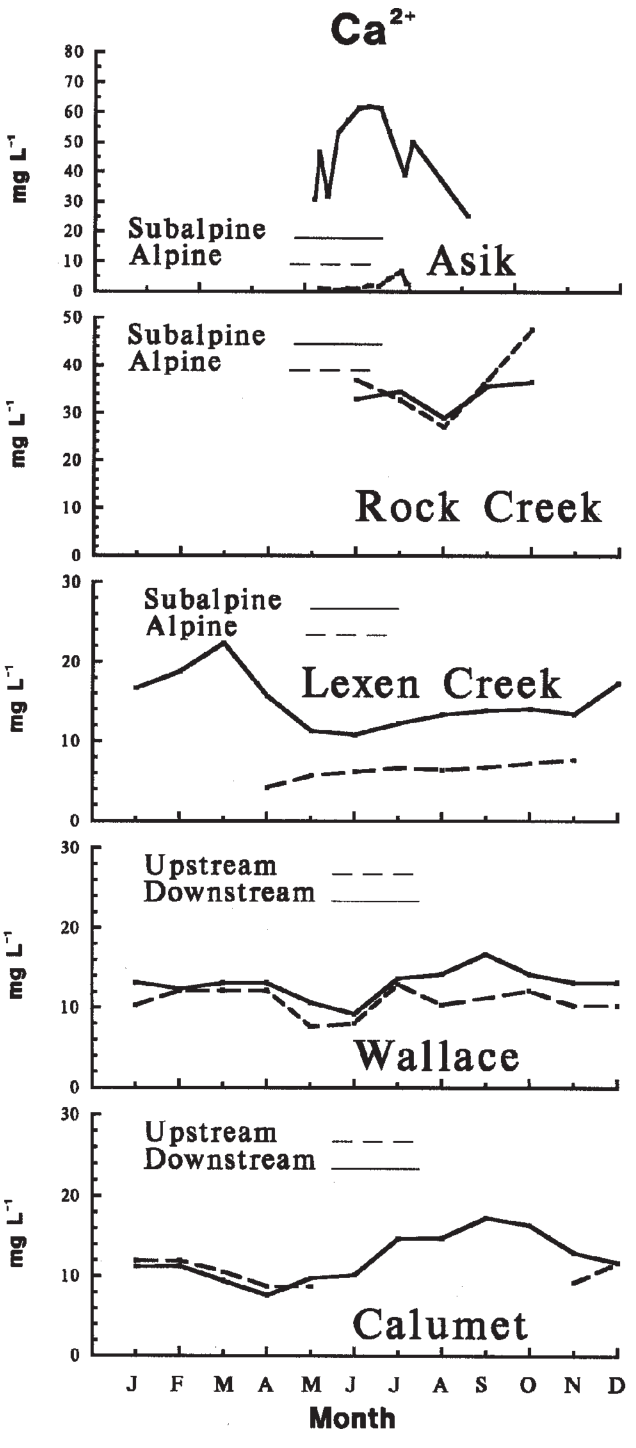

FIGURE 2. Seasonal change in stream-water $Ca^{2+}$ concentration, Calumet and Wallace Lake, Michigan; Lexen Creek, Colorado; Rock Creek and Asik watersheds, Alaska.

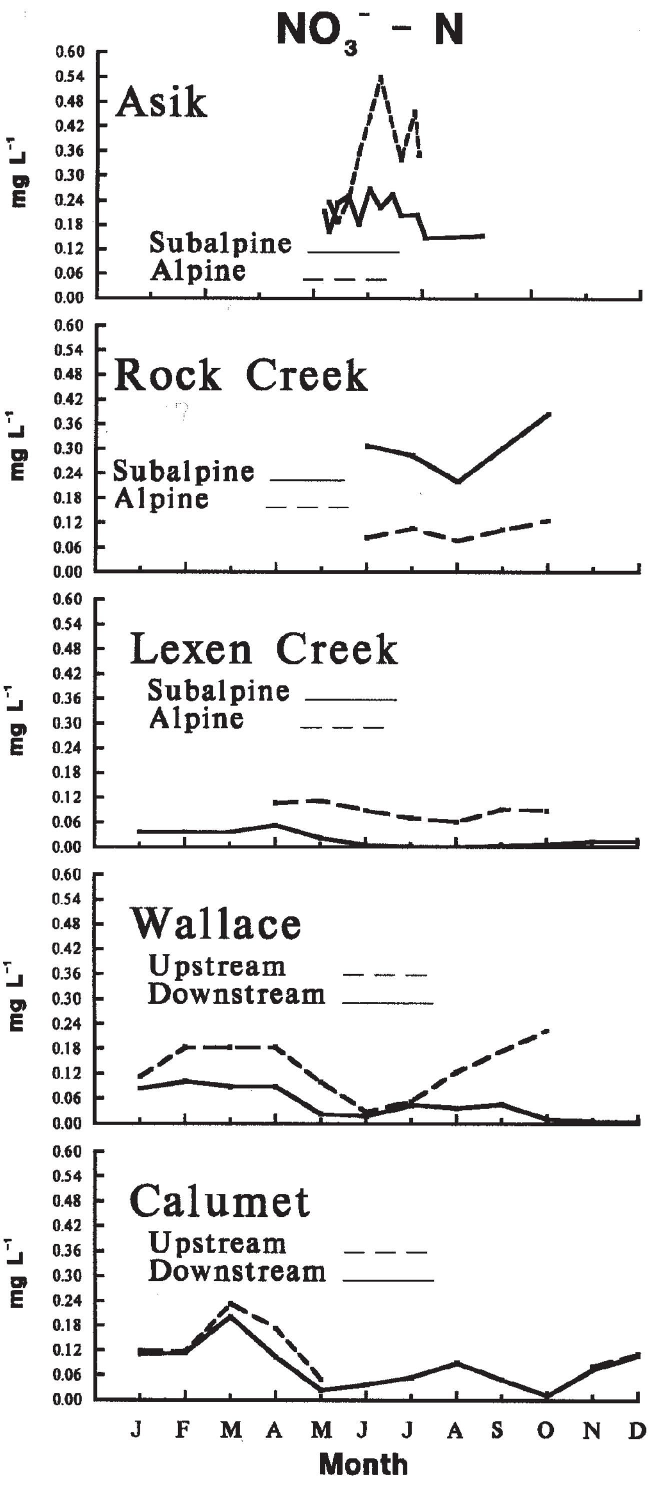

FIGURE 3. Seasonal change in streamwater $NO_3^-$ concentration, Calumet and Wallace Lake, Michigan; Lexen Creek, Colorado; Rock Creek and Asik watersheds, Alaska.

tionately increased $NO_3^-$ output[14]. The seasonal change in Asik alpine streamwater $Ca^{2+}$ and $NO_3^-$ concentrations differed from the subalpine in a pattern somewhat similar to that observed at Lexen (Figs. 2 and 3). The small change in $Ca^{2+}$ concentration during snowmelt in the Asik alpine suggests rapid entry and passage deep into the soil, resulting in a well-mixed pool of soil water reaching the stream. The seasonal change in subalpine streamwater $Ca^{2+}$ concentration indicated more contributions from shallow subsurface flow during and following snowmelt. The elevated Asik alpine streamwater $NO_3^-$ concentrations, especially during increased runoff (Figs. 1 and 3), also suggest rapid movement of inorganic N to below the rooting zone as in the Lexen Creek alpine.

Another potential factor affecting N output in some of these ecosystems was the presence of N-fixing vegetation. Alder was present in the Wallace watershed, in the subalpine of Rock Creek, and scattered in the Asik headwaters. Both Asik and Rock Creek had scattered patches of *Dryas* spp. in the alpine. At Wallace, differences in upstream and downstream $NO_3^-$ concentrations (Fig. 3) were, in part, attributed to the presence of alder (*Alnus rugosa*), which occupies a small percentage of the watershed area but generally is proximate to streams[20]. The seasonal forest-floor $NO_3^-$ pool sizes beneath alder were positively correlated ($p < 0.01$) with streamwater $NO_3^-$ output[8]. At Wallace, the inorganic N flux beneath the rooting zone of alder was 47% that in precipitation, or twice the amount at the watershed level. At Rock Creek, multiple upstream and downstream sampling stations showed an influence by alder (*A. crispa*) on subalpine stream inorganic N concentrations[19]. But alpine streamwater $NO_3^-$ concentrations were similar to the upstream levels in all other sites except Asik (Fig. 3). Patches of *Dryas* (*D. octopetala* and *D. integrifolia*) occur in the alpine of Rock Creek, but these species are not normally nodulated. At Asik, upstream $NO_3^-$ concentrations were about 70% higher than at the mouth of the watershed (Fig. 3), and DOC concentrations were equal from the alpine to the mouth into midsummer[7]. In the alpine, there were few N fixers except for scattered alder and *Dryas* spp. Alder in this watershed makes a significant contribution to soil N levels[21]; however, most or all alders were below the alpine streamwater sampling station. While the Asik alpine-soil organic pools were small relative to the subalpine, the high DOC export from the Asik alpine again suggested the importance of soil mineralization processes in dominating both the DOC and inorganic N export from that ecosystem.

In these ecosystems, soils were unfrozen except for the alpine of Lexen, Rock Creek, and Asik. Mitchell et al.[2], using long-term soil-temperature records from the USDA Forest Service Hubbard Brook Experimental Forest, New Hampshire, found elevated soil-water $NO_3^-$ concentrations and flux beneath the rooting zone following unusually cold periods and soil freezing. Deep soil frost, especially when associated with aboveground vegetation dieback, can sharply increase soil-water $NO_3^-$ flux beneath sugar maple[4]. Under laboratory conditions, freezing followed by thawing to temperatures from 3 to 14°C was found lethal to soil microflora, especially at the higher temperatures[22]. The alpine of Lexen, Rock Creek, and Asik typically have little snow cover because of high winds. This, coupled with porous, well-drained soils, the absence of canopy cover, and often favorable topographic orientation with the low angle of the sun in spring and early summer, results in rapid alpine soil warming to temperatures approaching 15°C[ 19].

## CONCLUSIONS

In these northern ecosystems, atmospheric N inputs were most strongly retained where inputs were highest. Almost all atmospheric $NH_4^+$ inputs were immobilized or transformed before reaching the stream. Only the northern treeline ecosystems had inorganic N outputs greater than inputs, and these ecosystems had the greatest proportion of total ecosystem N in the forest floor. $NO_3^-$ leaching below the forest-rooting zone showed no correlation with atmospheric inputs. However, two sites, Wallace and Rock Creek, showed a correlation between peak terrestrial net N mineralization rates and elevated streamwater $NO_3^-$ concentrations. Forest throughfall studies showed significant canopy retention of atmospheric inorganic N, especially where conifer snowfall interception and sublimation were large.

There was an inverse relationship between headwater stream $NO_3^-$ concentrations and atmospheric inorganic N inputs. Generally, headwater stream $NO_3^-$ concentrations were greater than at the mouth, independent of atmospheric inorganic N inputs. During snowmelt, headwater streamwater $Ca^{2+}$ and $NO_3^-$ concentrations showed a pattern of change that suggested rapid movement of meltwater and precipitation through porous soils. Preliminary evidence from budget studies suggests that headwater $NO_3^-$ flux is the result of climatic factors such as soil freeze–thaw processes coupled with large total N pools. At the watershed level, ecosystem inorganic N retention and release in these ecosystems appear regulated primarily by soil processes and hydrologic flowpath.

## ACKNOWLEDGMENTS

This research was primarily funded by the Long-term Watershed Research Program, National Park Service, and the U.S. Geological Survey Global Change Program. Many colleagues made this research possible through their support and ideas. Special thanks go to Drs. Charles Troendle, U.S. Forest Service (retired), Ft. Collins, CO; Raymond Herrmann, U.S. Geological Survey (retired), Ft. Collins, CO; Dan Binkley, Colorado State University, Ft. Collins; Charles Rhoades, University of Kentucky, Lexington; and Dan Markewitz, University of Georgia. Continuity and quality assurance in the field work have been provided by Dave Toczydlowski, Michigan Technological University, Houghton, MI, and Manual Martinez, U.S. Forest Service, Ft. Collins, CO. The laboratory analyses were conducted by Patricia Toczydlowski, Michigan Technological University, and Louise O'Deen, U.S. Forest Service, Ft. Collins, CO.

| | | |
|---|---|---|
| Received: | July | 27, 2001 |
| Revised: | October | 19, 2001 |
| Accepted: | October | 25, 2001 |
| Published: | November | 16, 2001 |

## BIOSKETCH

**Robert Stottlemyer** is a Research Scientist at the U.S. Geological Survey, Fort Collins, Colorado. His research interests include ecosystem structure and function, biogeochemical cycles, and long-term study of northern ecosystem structure and function. Primary research sites are located in U.S. reserves as National Parks and U.S. Forest Service Experimental Forests. Current research focuses on response of such systems to change in climate and nitrogen availability on ecosystem function especially in soils, and possible change in terrestrial export of dissolved organic carbon and nitrogen to the aquatic ecosystem.
